# Apelin Gene Therapy Increases Autophagy *via* Activation of Sirtuin 3 in Diabetic Heart

**DOI:** 10.17140/DROJ-1-115

**Published:** 2015-08-21

**Authors:** Xuwei Hou, Heng Zeng, Qin-Hui Tuo, Daun-Fang Liao, Jian-Xiong Chen

**Affiliations:** 1 Department of Pharmacology and Toxicology, University of Mississippi Medical Center, Jackson, MS 39216, USA; 2 Division of Stem Cell Regulation and Application, School of Integrated Chinese and Western Medicine, Hunan University of Chinese Medicine, Changsha, Hunan 410208, China

**Keywords:** Sirtuin 3, Autophagy, ROS, Apoptosis, Myocardial infarction, Diabetes

## Abstract

Heart failure is the leading cause of death in diabetic patients. Recently we showed that apelin gene therapy attenuates heart failure following myocardial infarction. This study further explored the potential mechanisms by which apelin may reduce cardiac injury in Postmyocardial infarction (MI)) model of diabetes. Wild type and Sirt3 knockout (Sirt3 KO) mice were induced into diabetes by intra-peritoneal (i.p.) Streptozotocin (STZ). STZ mice were then subjected to MI followed by immediate intramyocardial injection with Adenovirus-apelin (Ad-apelin). Ad-apelin treatment resulted in over expression of apelin in the ischemic hearts of STZ mice. Apelin over expression led to a significant increase in Sirt3 expression. Apelin over expression significantly reduced gp91^phox^ expression. This was accompanied by a significant reduction of reactive oxygen species formation. Ad-apelin treatment also dramatically reduced NF-κb-p65 expression in WT-STZ mice. Over expression of apelin further enhanced autophagy markers (LC3-II and beclin-1) expression in post-MI heart. Most intriguingly, knockout of Sirt3 in STZ mice abolished these beneficial effects of apelin treatment. *In vitro*, knockout of Sirt3 in EPCs significantly enhanced high glucose-induced ROS formation. Conversely, treatment of Sirt3 KO-EPCs with NADPH oxidase inhibitor led to two fold increase in LC3-II levels. Our studies demonstrate that apelin increases autophagy *via* up regulation of Sirt3 and suppression of ROS-NF-κb pathway in diabetic heart.

## INTRODUCTION

Long known to augment the risk for cardiovascular disease, Diabetes Mellitus (DM) increases mortality of patients with Heart Failure (HF) over that observed in HF patients without DM.^1^ Cardiovascular disease is one of the major complications of DM.^2^ Clinical studies show that Myocardial Infarction (MI) is the leading cause of morbidity and mortality in the patients with DM.^3,4^ A population-based study also reveals that the incidence of MI in diabetic patients is significantly higher than non-diabetic patients.^5^ Therefore, it is urgent to develop new agents for the treatment of post-MI heart failure in DM.

Apelin is a bio-activated peptide which binding to the apelin receptor (APJ).^6^ Apelin has been shown to protect the heart against ischemia injury and reduce infarct size.^7^ A recent study also showed that deficiency of apelin exacerbated ischemia-reperfusion injury.^8^ We have reported that over expression of apelin promoted myocardial angiogenesis and improved cardiac function in post-MI diabetic STZ mice and these beneficial effects of apelin were mediated through activation of Sirt3.^9^ Sirt3 is a member of a highly conserved family of protein deacetylases, which is closely associated with the prolonged lifespan of human.^10^ Sirt3 has been shown to regulate cardiomyoctye apoptosis, survival and cardiac hypertrophy.^11^ Previously, we also observed that treatment with Bone Marrow Cells (BMCs) over-expressing apelin enhanced myocardial angiogenesis and functional recovery, accompanied by increased Sirt3 levels in the ischemic heart.^12^ Our study further showed that knockout of Sirt3 blunted the protective effect of apelin in cultured EPCs.^12^ These findings indicate a critical role of Sirt3 in apelin-mediated protective effect in post-MI heart.

The present study was designed to evaluate the functional role of Sirt3 in apelin-mediated beneficial effects against ischemic injury in diabetic mouse model. Wild type and Sirt3 knockout (Sirt3 KO) mice were treated with streptozotocin (STZ) to induced hyperglycemic DM model followed by myocardial infarction by ligation of Left Anterior Descendant (LAD) artery. Using this ischemic STZ mouse model, we have examined the effects of apelin gene therapy on the autophagy and ROS formation in ischemic hearts of diabetes. Moreover, we have explored the potential mechanisms by which apelin regulates myocardial autophagy in diabetes.

## MATERIALS AND METHODS

All procedures conformed with the Institute for Laboratory Animal Research Guide for the Care and Use of Laboratory Animals and were approved by the Animal Care and Use Committee of University of Mississippi Medical Center (protocol identifier: 1280). The investigation conformed to the National Institutes of Health Guide for the Care and Use of Laboratory Animals (NIH Pub. No. 85-23, Revised 1996).

### Experimental Animal Model Treatment

Wild type control and Sirt3 knockout (Sirt3 KO) mice (obtained from Jackson laboratory, Bar Harbor, Maine, USA) were bred by our lab. Experimental mice (male at 4–5 month age) were intra-peritoneal (i.p) injected with streptozotocin (STZ, 50 mg/kg, Sigma Co, MO, USA) for 5 days to induce diabetes. At 6 weeks, mice with blood glucose level >300 mg/dl were selected for the left anterior descending coronary (LAD) artery ligation to induce MI. Experimental STZ mice were anesthetized with ketamine (100 mg/kg) plus xylazine (15 mg/kg), intubated, and artificially ventilated with room air. A left thoracotomy was performed, and the LAD was exposed and ligated with 8-0 nylon suture. Ischemic areas were intramyocardial injected with adenovirus-apelin (Ad-apelin) and adenovirus-β-gal adenovirus (Ad-β-gal) at the dose of 1×10^9^ PFU per heart at four sites. Experimental STZ mice were divided into 4 groups: (i) WT-STZ+Ad-β-gal (n=14 mice); (ii) WT-STZ+Ad-apelin (n=14 mice); (iii) SIRT3KO-STZ+Ad-β-gal (n=14 mice); and SIRT 3KO-STZ+Ad-apelin (n=14 mice). After 2 weeks of ad-apelin or Ad-β-gal gene therapy, mice were sacrificed by cervical dislocation under anesthesia with isoflurane.

### Western Blot Analysis of PHD2, HIF-1α, NF-κb, Apelin, gp91^phox^, Beclin-1 and LC3-I/II Expression

Hearts were harvested and homogenized in lysis buffer for Western analysis. Following immunoblotting, the membranes were blotted with HIF1-α, apelin, gp91^phox^ (1:1000, Cell Signaling, MA, USA), PHD2, NF-κb-p65, beclin-1 and LC3-I/II (1:1000, Santa Cruz, CA, USA) antibodies. The membranes were then washed and incubated with a secondary. Antibody coupled to horseradish peroxidase and densitometric analysis was carried out using image acquisition and analysis software (TINA 2.0).

### ROS Formation Assays in Heart Tissue

To detect *in situ* generation of ROS in heart tissues, Dihydroethidium (DHE) staining was performed. DHE (1 nM, Molecular Probes, Oregon, USA) was applied to each heart tissue section and cover-slipped. Slides were incubated in a dark, humidified chamber at 37 °C for 30 min. The nuclei were counterstained with 4,6-diamino-2-phenyl indole (DAPI). The relative density of red (DHE) fluorescence was quantified by measuring 5 random fields per section using image-analysis software (Image J, NIH).

### Endothelial Cell Progenitor Isolation, Culture and Identification

EPC was isolated and cultured from femur and tibia bone marrow of WT and Sirt3 KO mice as described previously.^13^ Two EPC markers, IB4 (1:50 dilute) and CD34 (1:200 dilute), were used for EPC identification by immunohistochemistry.

### EPC Treatment and Transfection

To mimic *in vivo* hyperglycemic conditions of DM model, EPC were exposed to high glucose (30 mmol/L) for 24 hours, and followed by transfection with Ad-apelin and Ad-β-gal (1×10^9^ PFU) in serum-free medium. An Ad-green fluorescent protein (Ad-GFP) was applied to the cultured EPC as a marker to determine the transfection efficiency before transfected with Ad-apelin and Ad-β-gal.

To detect the intra-cellular ROS production in EPC, 1×10^4^ cells were seeded in chamber wells and cultured for 24 hours to reach >80% confluence. Then CM-H2DCFDA (10 μmol/L, Molecular Probes, Oregon, USA) was added to chamber wells for 30 minutes. The nuclei were counterstained with 4,6-Diamino-2-phenyl indole (DAPI). The relative density of green (DCFDA) fluorescence was quantified by measuring 5 random fields per section using image-analysis software (Image J, NIH).

### Statistical Analysis

Data are presented as the mean±standard deviation. Statistical analysis of data were performed with one-way ANOVA followed by the post hoc test and P values less than 0.05 were considered as significant.

## RESULTS

### Apelin Gene Therapy Increases Post-Mi Survival Rate of Mouse

WT-STZ mice receiving Ad-apelin or Ad-β-gal treatment were survived for 2 weeks of post-MI. There was no death in Sirt3 KO-STZ mice received Ad-apelin treatment after 2 weeks of post-MI, whereas, there was an approximately 42.9% death rate in Sirt3 KO-STZ mice received Ad-β-gal treatment (P<0.001).

### Apelin Gene Therapy Upregulates Sirt3 Expression in the Hearts

**Figure 1:**
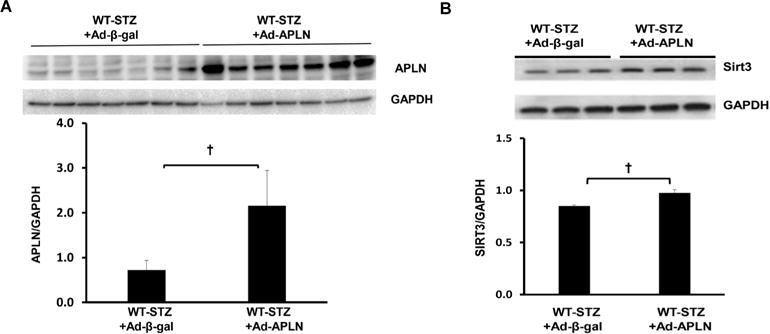
Apelin gene therapy on apelin and Sirt3 expression in post-MI STZ mice. (A) Ad-apelin treatment significantly increased apelin expression in the heart of WT-STZ (n=3, †P<0.05). (B) WT-STZ mice had a significant increase in Sirt3 expression in the heart after Ad-apelin gene therapy (n=3, †P<0.05)

As shown in Figure 1A, intramyocardial injection with Ad-apelin led to apelin overexpression in the hearts of WT-STZ mice. Sirt3 expression was significantly upregulated by apelin overexpression in the hearts of WT-STZ mice compared to WT-STZ mice treated with Ad-β-gal (Figure 1B).

### Apelin Gene Therapy Attenuates Myocardial gp91^phox^ Expression and ROS Formation in Post-MI STZ Mice

**Figure 2:**
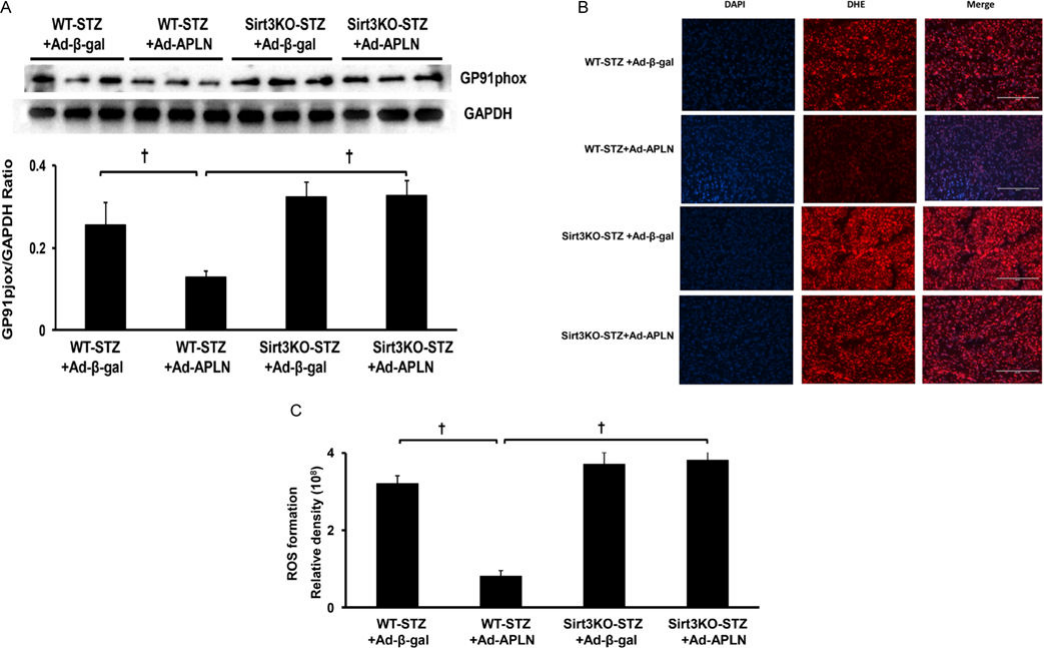
Apelin gene therapy on myocardial ROS formation. (A) apelin gene therapy significantly reduced gp91phox expression compared to WT-STZ mice treated with Ad-β-gal. Knockout of Sirt3 blunted apelin gene therapy-mediated gp91phox expression in Sirt3 KO-STZ mice (n=3 mice, †P<0.001). (B and C) DHE staining of ROS formation shows that Ad-apelin treatment dramatically inhibited ROS formation in the heart of WT-STZ mice (n=5, †P<0.001). Ad-apelin treatment had little effect on the ROS formation level in Sirt3 KO-STZ mice. WT-STZ mice treated with Ad-apelin had a lower ROS formation level than Sirt3KO-STZ, but did not reach significant difference. The ROS formation levels were similar between mice received Ad-apelin and Ad-β-gal treatment in Sirt3KO-STZ mice.

Ad-apelin treatment significantly inhibited NADPH oxidase gp91^phox^ expression in post-MI STZ mice, but failed to reduce gp91^phox^ expression in post-MI Sirt3 KO mice (Figure 2A). Ad-apelin treatment significantly reduced ROS formation in the hearts of WT-STZ mice when compared with Ad-β-gal treatment. In Sirt3 KO-STZ mice, Ad-apelin treatment did not suppress ROS formation in comparison with Ad-β-gal treatment (Figures 2B and 2C).

### Apelin Gene Therapy Elevates Autophagy Gene Beclin-1 and LC3-II Levels in Post-MI STZ Mice

**Figure 3:**
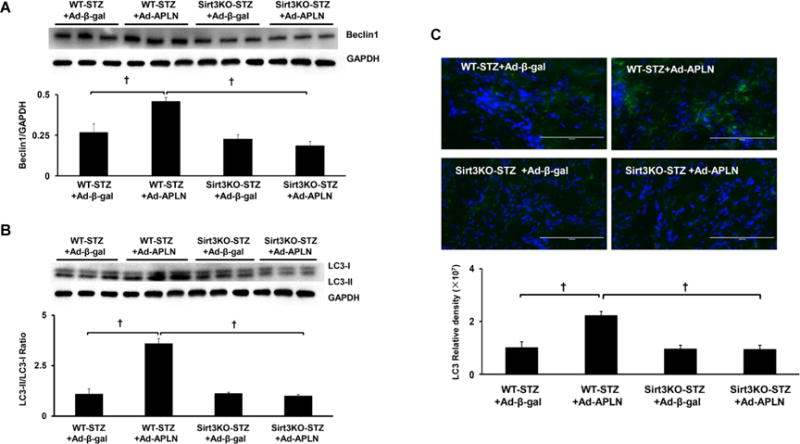
Apelin gene therapy on myocardial autophagy. (A) Apelin overexpression significantly upregulated beclin-1 expression compared to WT-STZ mice treated with Ad-β-gal. Knockout of Sirt3 blunted apelin gene therapy-mediated beclin-1 expression in Sirt3 KO-STZ mice (n=3 mice, †P<0.001). (B) Apelin gene therapy significantly enhanced LC3-II expression compared to WT-STZ mice treated with Ad-β-gal. Knockout of Sirt3 blunted apelin-mediated LC3-II expression in Sirt3 KO-STZ mice (n=3 mice, †P<0.001). (C) Immuno-staining of LC3-II levels in the hearts. Measurement of fluorescent density in the heart section showed that Ad-apelin treatment dramatically increased the LC3-II levels compared to Ad-β-gal injection In WT-STZ+MI mice. In contrast, the Ad-apelin and Ad-β-gal treatment did not change the LC3-II levels in Sirt3 KO-STZ+MI mice.

Overexpression of apelin resulted in significant increases in beclin-1 and LC3-II expression in WT-STZ+MI mice when compared with Ad-β-gal treatment (Figures 3A and 3B). No significant alterations in beclin-1 or LC3-II expression were found in Sirt3 KO-STZ+MI mice received Ad-apelin treatment (Figures 3A and 3B). Using immunohistochemistry staining, we further confirmed that apelin overexpression dramatically increased number of LC3-II positive cells compared to Ad-β-gal treatment. In contrast, the Ad-apelin treatment did not change LC3-II levels in Sirt3 KO-STZ+MI mice (Figure 3C).

### Apelin Gene Therapy Attenuates NFKb Expression, but not Prolyl Hydroxylase-2 (PHD2) and HIF-1α Expression

**Figure 4:**
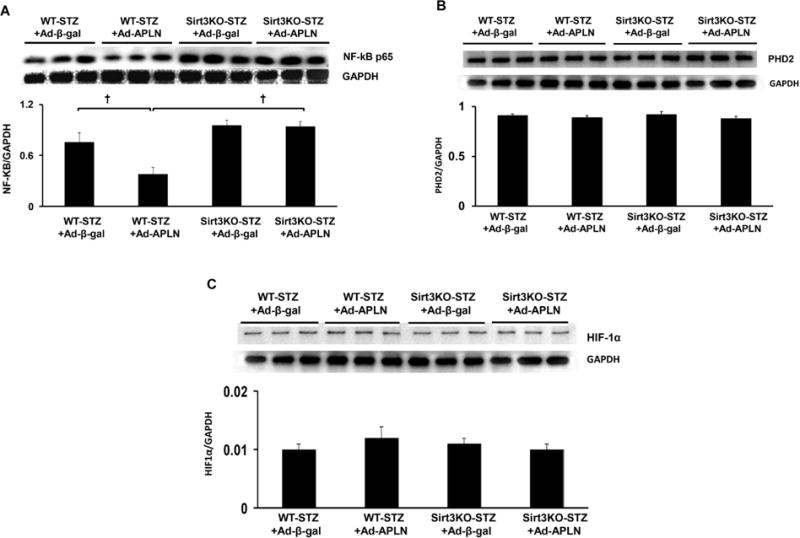
Apelin gene therapy on NF-kb and HIF-1α expression. (A) Ad-apelin treatment significantly reduced NFkb expression in WT-STZ post-MI mice in comparison with Ad-β-gal injection. Apelin gene therapy had little effects on the NFkb expression in Sirt3 KO-STZ mice. (B and C) Ad-apelin treatment did not alter HIF-1α and PHD2 levels in WT-STZ and Sirt3 KO-STZ post-MI mice in comparison with Ad-β-gal injection.

To determine whether apelin regulates the transcriptional regulator gene expression, NFKb-p65 and PHD2/HIF-1a expression were examined. As shown in Figure 4A, the NFKb expression level was significantly higher in post-MI Sirt3 KO-STZ mice. Overexpression of apelin significantly reduced NFKb-p65 expression level in post-MI WT-STZ mice, but failed to inhibit its expression in post-MI Sirt3 KO-STZ mice. Moreover, apelin overexpression had little effects on HIF-1α and PHD2 expression both in WT and Sirt3 KO-STZ mice (Figures 4B and 4C).

### Overexpression of apelin reduces ROS formation in EPC

**Figure 5:**
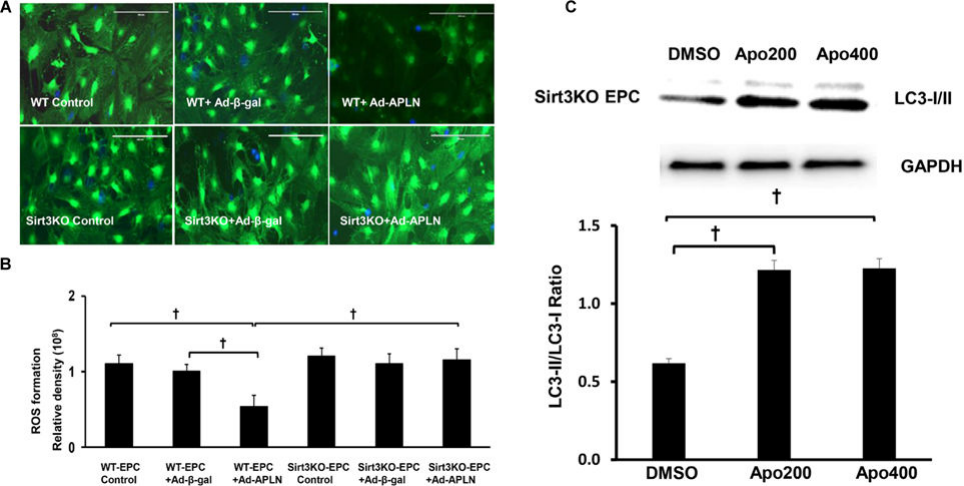
Knockout of Sirt3 abolishes apelin induced suppression of ROS formation in EPC. (A) Representative images of overexpression of apelin on ROS formation in EPC under High Glucose (HG) conditions *in vitro*. (B) Overexpression of Ad-apelin in EPC attenuated HG-induced ROS formation (n=5, †P<0.001). The inhibitory effect of apelin on HG-induced ROS formation was abolished in Sirt3 KO-EPC. (C) Treatment of Sirt3 KO-EPC with apo (200 and 400 μM) enhanced LC-3 II expression.

To mimic STZ hyperglycemic condition *in vivo*, cultured EPCs were exposed to high glucose (30 mmol/L) for 24 hours before transfection with Ad-apelin. Overexpression of apelin significantly reduced high glucose induced ROS formation. Moreover, Knockout of Sirt3 abolished apelin-mediated suppression of ROS formation in EPC under high glucose conditions (Figures 5A and 5B). To determine the interactions of Sirt3, NADPH oxidase and ROS on autophagy gene expression, Sirt3KO-EPCs were exposed to NADPH oxidase inhibitor apocynin for 24 hours. As shown in Figure 5C, treatment of Sirt3KO-EPCs with Apo (200 and 400 microM) increased LC3-II expression.

## DISCUSSION

Our previous studies demonstrated that SIR3 plays a central role in the apelin-mediated cardiac protection. We have shown that: (a) Sirt3 is essential for apelin-induced angiogenesis in response to ischemia in diabetes;^9^ (b) loss of Sirt3 limits apelin-overexpression bone marrow cell-mediated angiogenesis and cardiac repair;^12^ and (c) upregulation of Sirt3 by overexpression of apelin ameliorates diabetic cardiomyopathy in diabetic db/db mice.^14^ In this study, we further demonstrated that apelin overexpression increases autophagy and reduces NADPH oxidase derived ROS formation in the ischemic hearts of diabetes. We also revealed that Sirt3 has a critical role in apelin-induced autophagy and suppression of ROS formation. Our present findings elucidate a novel mechanism by which apelin protects the ischemic heart of diabetes.

Autophagy is a highly conserved cellular process that maintains cell survival by releasing energy substrates *via* the lysosome-dependent pathway and by removing defective organelles.^15^ Accumulating evidence also indicate that autophagy plays a key role for the maintenance of cardiomyocytes structure and function under ischemia and pressure-overload.^16–19^ Enhancing autophagy protects against ischemia/reperfusion injury in cardiac myocytes.^20^ Augmenting autophagy is emerging as a therapeutic strategy for acute myocardial ischemia.^21^ In the present study, we showed that overexpression of apelin increased the expressions of two important autophagy markers, beclin-1 and LC-3II, in the ischemic heart. Intriguingly, knockout of Sirt3 abrogated this effect, suggesting that induction of autophagy through activation of Sirt3 may contribute to the protective action of apelin against ischemic injury.

NADPH oxidase subunit gp91^phox^, known also as Nox2, is an enzyme solely dedicated to the generation of ROS.^22^ Gp91^phox^ expression is upregulated in the ischemic heart and salvages cardiomyocytes from ROS-induced injury.^23^ Knockdown of Nox2-NADPH oxidase using siRNA improves cardiac function following myocardial infarction.^24^ Loss of NOX2 (gp91^phox^) further reduces oxidative stress and prevents progression to advanced heart failure.^25^ In this study, we showed that the apelin overexpression significantly inhibited gp91^phox^ expression in heart of STZ mice. This was accompanied by reduced ROS formation and increased autophagy gene expression. In contrast, knockout of Sirt3 in STZ mice completely abolished these beneficial effects of apelin gene therapy. As one of the major mitochondrial NAD+-dependent deacetylase, Sirt3 is involved in mitochondrial ROS formation.^26,27^ Sirt3 regulates ROS production by directly binding and deacetylating mitochondrial complex I and II.^28^ Sirt3 has been shown to attenuate hydrogen peroxide-induced oxidative stress through the preservation of mitochondrial function.^29^ NADPH oxidase has been shown involved in autophagy activation in cardiomyocyte.^30^ Previously, we have shown that loss of Sirt3 enhanced ROS formation and apoptosis in EPCs.^31^ Consistent with these findings, we showed that knockout of SIRT3 abolished apelin-mediated suppression of ROS formation in heart tissue and cultured EPCs. Moreover, pharmacological inhibition of NADPH oxidase in Sirt3 KO-EPCs increased LC-3II expression. These data suggest a possible interaction among Sirt3, gp91^phox^ and ROS in the regulation of autophagy.

NF-κb and HIF-1α are two important transcriptional factors involved in the regulation of autophagy gene expression.^32,33^ ROS-mediated NF-κb activation has been shown to lead to the autophagic degradation in fibroblasts.^34^ Our data showed that apelin gene therapy significantly suppressed NF-κb expression, but has little effect on HIF-1α and its regulator PHD2 levels. Furthermore, knockout of Sirt3 abrogated apelin-induced suppression of NF-κb activation and failed to affect autophagy gene expression in ischemic heart of STZ mice. These data implicate that apelin may upregulate autophagy in infarcted heart of diabetic mice *via* suppression of NF-κb, rather that HIF-1α. We therefore hypothesized that deficiency (loss) of Sirt3 in diabetic heart may cause gp91^phox^ activation and increase ROS formation, which subsequently inhibits autophagy expression *via* activation of NF-κb. However, further study is warranted to clarify this signaling pathway.

In summary, the current study provides direct evidence that overexpression of apelin reduces ROS formation and enhances autophagy *via* upregulation of Sirt3. Our data suggest that modification of Sirt3 with apelin could be used as a novel therapy strategy for the treatment of diabetes-associated heart failure.

## ABBREVIATIONS

HF: Heart Failure
DM: Diabetes Mellitus
BMCs: Bone Marrow Cells
STZ: Streptozotocin
LAD: Left anterior descendant artery
DHE: Dihydroethidium
Ad-GFP: Ad-green fluorescent protein
DAPI: Diamino-2-phenyl indole
MI: Myocardial Infarction
